# Perceived economic inequality is linked to poorer sleep quality

**DOI:** 10.1186/s40359-025-03405-5

**Published:** 2025-10-01

**Authors:** Wenxuan Liu, Qinglu Wu, Yuebin Xu, Nicolas Sommet, Hongfei Du

**Affiliations:** 1 Department of Psychology, Beijing Normal University at Zhuhai, Zhuhai, China; 2 Beijing Key Laboratory of Applied Experimental Psychology, National Demonstration Center for Experimental Psychology Education (Beijing Normal University), Faculty of Psychology, Beijing Normal University, Beijing, China; 3 Institute of Advanced Studies in Humanities and Social Sciences, Beijing Normal University, Zhuhai, China; 4 Swiss National Centre of Competence in Research LIVES, University of Lausanne, Lausanne, Switzerland; 5 Center for Studies of Psychological Application, South China Normal University, Guangzhou, China

**Keywords:** Perceived economic inequality, Sleep quality, Social comparison, Stress

## Abstract

Economic inequality is believed to negatively affect individuals’ psychological and physical health. We hypothesized that perceptions of greater economic inequality predict poorer sleep quality through social comparison and stress. We tested this hypothesis across three studies. Study 1 used a large-scale longitudinal dataset with a nationally representative sample in China (*n* = 37,984) to investigate the within-participant association between economic inequality and sleep quality over time. A fixed-effects model revealed that participants perceiving increasing economic inequality reported poorer sleep quality, whereas objective economic inequality did not predict sleep quality. Study 2 used a cross-sectional college sample (*n* = 668) and a cross-sectional community sample (*n* = 1,009) to replicate the findings and further examine psychological mechanisms underlying the relationship between perceived economic inequality and sleep quality. Structural equation models confirmed that higher perceived economic inequality was associated with poorer sleep quality. Moreover, in both samples, the relationship was sequentially mediated by upward social comparison and stress. These findings provide important insights into understanding the psychological and epidemiological implications of economic inequality.

**Supplementary Information:**

The online version contains supplementary material available at 10.1186/s40359-025-03405-5.

## Introduction

Sleep plays a vital role in life and health throughout biological evolution [1], as evidenced by robust associations between sleep and various health outcomes [2, 3]. Despite this, sleep disorders remain a prevalent issue globally [4]. A crucial and urgent issue is understanding the socioeconomic determinants of sleep quality [5].

Considerable research has focused on how individuals’ socioeconomic conditions influence sleep, revealing that people with low socioeconomic status (SES) experience poorer sleep quality, shorter sleep durations, and higher rates of insomnia [6–8].

Recognizing that people are simultaneously embedded within diverse socio-economic contexts characterized by varying degrees of inequality, our research adopts a different perspective by exploring how the broader socioeconomic context, specifically economic inequality, may relate to sleep. Building on past decades of research that explores the health implications of economic inequality [9], the current research aims to further understand whether and how economic inequality is associated with sleep quality. The results will be useful in advancing our knowledge of the socio-economic determinants of sleep quality, and in adopting a socio-economic perspective in developing interventions to reduce sleep issues.

### Does economic inequality affect sleep?

Economic inequality is a major social challenge. Over the last two decades, the gap between rich and poor has widened for most countries and for 70% of the world’s population [10]. Theories suggest that exposure to economic inequality may create more social comparison or status anxiety, potentially taking a toll on mental health [11–13]. Meta-analyses have demonstrated a negative association between economic inequality and mental health conditions, but the magnitude of these association is very small [14, 15]. However, focusing on economic inequality and sleep might be more informative. Sleep is often used as an indicator of mental health [16], given its sensitivity to stress and anxiety [17].

To the best of our knowledge, only five studies investigated the link between economic inequality and sleep, four of which focused on sleep duration as an outcome and yielded mixed findings. Some studies showed a negative association between higher inequality and sleep duration [18, 19], others reported a mix of negative and null associations [20], and one study even found a positive correlation between economic inequality and sleep duration [21]. These mixed finding may be due to the complex nature of sleep duration as a health indicator, as both short and long sleep duration could be risk factors for health [22]. Researchers suggest that sleep quality may be a stronger predictor of health outcomes than sleep duration [23]. To date, Clément et al. [24] is the only study that has directly examined sleep quality, finding a negative association with economic inequality. However, their results were not entirely conclusive, as the negative relationship was only observed among women, not men.

Additionally, all existing studies on inequality and sleep have focused objective economic inequality, defined as the unequal distribution of income or wealth in a given area (e.g., country, state, city). They have overlooked the potential influence of perceived economic inequality, which reflects individuals’ perceptions of unequal distributions and may differ from actual levels [25, 26]. Objective economic inequality may not have a uniform impact on all people living in the same area, while perceived economic inequality tends to be a more robust predictor of psychological outcomes [12, 27].

Given the limited evidence and inconsistent findings regarding the link between economic inequality and sleep, the current research will primarily focus on the role of perceived economic inequality, while using sleep quality rather than sleep duration as the outcome variable. While we tentatively hypothesize that objective economic inequality may negatively predict sleep quality, we are more assertive regarding the effect of perceived economic inequality:

#### Hypothesis 1

Both objective (Hypothesis 1a) and perceived (Hypothesis 1b) economic inequality are associated with poorer sleep quality.

### Social comparison and stress as psychological mechanisms

Little is known about the psychological mechanisms underlying the relationship between economic inequality and sleep. This study also aims to investigate two potential mechanisms: social comparison and stress.

### Economic inequality and upward social comparison

Social comparison theory posits that individuals have a tendency to compare themselves with others, particularly those perceived as higher in social status (i.e., the unidirectional upward drive [28]). Economic inequality increases the salience of economic segmentation, making economic differences between the rich and the poor more readily apparent in the environment [29]. Residing in a stratified place (or perceiving living in such a place) encourages people to categorize their social world into the “haves” and the “have-nots” [30], to place greater value on interindividual competition as well as personal success and achievement [31, 32], and to ascribe more importance to relative income [33]. This dynamic fuels a culture of social comparison, where people are concerned about knowing their place and that of others in the economic hierarchy [34, 35]. More importantly, Duesenberry’s relative income hypothesis [36] suggests that consumption is more likely influenced by relative income, that is, individuals consider their income in comparison to others when making consumption decisions. It is particularly driven by unfavorable social comparison (i.e., upward social comparison). In other word, when people place greater importance on status or relative income, they tend to engage more in upward social comparison.

Moreover, perceptions of inequality are deeply rooted in upward social comparison processes [37]. Perceived economic inequality leads individuals to feel less wealthy compared to others [38], and arouses a sense of relative deprivation [39], both of which are closely linked to upward social comparison. Payne et al. [40] found that higher perceived economic inequality led people to exhibit stronger desires to satisfy their needs and a greater willingness to take financial risks. In this research, this increase in risk-taking behavior was driven by a heightened salience of upward comparison. In essence, both objective and subjective economic inequality foster upward social comparison, thereby shaping individuals’ psychological outcomes and behaviors.

### Economic inequality and stress

Stress may provide another explanation for the link between economic inequality and sleep quality. The status anxiety hypothesis suggests that inequality can increase worry by amplifying concerns about falling behind or being seen as undeserving [13, 41] (but see Walasek and Brown [42]). Furthermore, the sense of status threat produced by economic inequality could be a powerful stressor and ultimately weigh on mental health [43]. Additionally, from a neurobiological perspective, sleep is partly regulated by the serotonin system, which is also involved in emotion and stress regulation [44]. This suggests that stress has the potential to disrupt normal sleep patterns.

### The chain mediation of upward social comparison and stress

Upward social comparison has long been established as a stressor [45] and is known to trigger negative emotions [46]. Upward social comparison is thought to be linked to ruminative thoughts [47] that negatively affect self-esteem and can engender depression and anxiety [48, 49]. Therefore, individuals who perceive greater economic inequality may be more prone to engage in upward social comparison, which may further generate stress and manifest in poorer sleep quality. Given that the current research primally focuses on perceived economic inequality and sleep, we propose the following hypothesis:

#### Hypothesis 2

The negative association between perceived economic inequality and sleep quality is serially mediated by upward social comparison and stress.

## Overview of the current research

The current research comprises three studies to investigate the link between economic inequality and sleep quality in China, where both issues have become increasingly prominent in recent years [50, 51]. Study 1 used a longitudinal dataset to test whether objective and perceived economic inequality predicts sleep quality within participants over time. Study 2 used a sample of college students (Study 2a) and a sample of community residents (Study 2b) to replicate Study 1’s findings and examined the mediating roles of upward social comparison and stress. It is worth noting that the COVID-19 pandemic, which overlapped with our data collection periods, has been shown to exacerbate economic inequalities by disproportionately affecting vulnerable populations. Families and regions more severely impacted by the pandemic experienced greater income losses [52, 53]. Concurrently, the Chinese government’s announcement of the “common prosperity” policy in 2021 explicitly acknowledged concerns about wealth disparity and signaled intentions to address economic inequality [54]. These contextual factors may have influenced how participants perceived economic inequality during our study period, providing important background for interpreting our findings. Complete materials, raw data (or instructions to retrieve the secondary survey data), and scripts reproducing the findings are available via the OSF: https://osf.io/n3fg9/?view_only=49ed37edd68849d78a95a9231fe9952c.

## Study 1

Study 1 used a nationally representative longitudinal dataset in China to test objective economic inequality and perceived economic inequality as predictors of sleep quality (Hypothesis 1).

### Method

#### Participants

The data were sourced from the China Family Panel Studies (CFPS), a project managed by the Institute of Social Science Survey of Peking University (http://www.isss.pku.edu.cn/cfps/). The CFPS is a nationwide, large-scale survey covering 25 of the 34 Chinese provinces and it has been conducted every two years since 2010. The CFPS employs a novel rural-urban, integrated, multi-stage probability-proportion-to-size (PPS) sampling scheme with implicit stratification to ensure the validity and representativeness of its sample [55]. We used the data from four waves: 2012 (T1), 2016 (T2), 2018 (T3), and 2020 (T4), since these waves were the only ones that included measures of both perceived economic inequality and sleep quality. The final sample included 33,122 individuals with wave-specific participation as follows: 25,078 in T1, 28,429 in T2, 27,169 in T3, and 19,860 in T4^1^.

The average age of respondents was 46.15 (*SD* = 16.24) and half were female. Regarding education, 75.19% of respondents had received some levels of education, with 23.17% completing high school. To ensure accurate representation of real purchasing power and balanced comparison across households of different sizes, we adjusted respondents’ yearly income to 2020 CNY levels, using OECD’s square root-based equivalization procedure (https://www.oecd.org/els/soc/OECD-Note-EquivalenceScales.pdf). The median equivalized annual household income, adjusted for inflation, was 60,001 CNY (approximately 8,202 USD). Study 1 used publicly available de-identified secondary data and the authors had no interaction with the participants; therefore, human subjects ethical review was not required.

#### Measures

The descriptive statistics for the focal variables can be found in Table S1.

**Province Objective Economic Inequality** was assessed using the Gini coefficient. Given that participants were nested in 25 provinces across four waves, we calculated a Gini coefficient using annual household income for each of the 25 × 4 = 100 province-wave units [56]. The CFPS included an average of 540 households sampled per province across the years (for a description of the household sample size in each province and the specific Gini coefficients for each province-wave, see *Supplementary Materials*). While the within-year, within-province samples could ideally be larger, they represent the most representative income data currently available at the provincial level in China. The Gini coefficient can range from 0, indicating perfect equality (where everyone in a given province during a given year has the same income), to 1, indicating perfect inequality (where one person in a given province during a given year has all the income and everyone else has none).

**Individual Perceived Economic Inequality** was assessed using the following item: “How severe do you think the economic inequality in our country is” [57]. This measure has been used as a measure of perceived economic inequality in previous research [25, 57]. Participants responded on an 11-point scale from 0 = *not severe at all* to 10 = *very severe*, with higher scores indicating higher perceived levels of inequality.

**Individual Sleep Quality** was assessed using a single item extracted from the Center for Epidemiologic Studies Depression (CES-D) Scale [58]: “During the past week my sleep was restless.” This measure is commonly used to assess sleep quality [59, 60]. Participants responded on a 4-point scale from 1 = *almost never (less than one day)* to 4 = *most of the time (5–7 days)*. Reverse scoring was applied, with higher scores indicating better sleep quality.

**Country-Level and Individual-Level Sociodemographic Covariates.** At the country level, we controlled for the level of economic development across different provinces. Specifically, gross domestic product (GDP) per capita was obtained from the National Bureau of Statistics of China (https://data.stats.gov.cn/index.htm) and served as an annual indicator of each province’s level of development. At the individual level, we controlled for the following demographic variables: respondents’ age, education (0 = *uneducated/informally educated*, 1 = *formally educated*), and household annual income.

#### Statistical strategy

We used fixed-effects *panel* model^2^ to test our hypotheses, which allowed us to estimate the pooled within-participant effects of objective and perceived economic inequality over the life course net of period effects. In simpler terms, we only focus on how objective and perceived inequality changes predict within-participant’s sleep quality, not the differences between participants. We included province fixed effects to account for the fact that different participants may belong to the same province. We also applied standard errors adjusted for both participant and province clustering to address the issue of nonindependence of residuals in longitudinal designs. The fixed-effect panel model regression equation is presented in the following equation:

Sleep Quality_ijt_ = β_0_ + β_1_ Perceived Inequality_ijt_ + β_2_ Gini_ijt_ + β_3_ GDP per capita _ijt_  + β_4_ Age_ijt_ *+* β_5_ Education_ijt_ *+* β_6_ Income_ijt_ +β_7_ Year_ijt_  *+* α_*i*_  *+* λ_*j*_  *+ u*_*ijt*_ (1).

*…i* = 1, 2, …, 33,122 participants, *j* = 1, 2, …, 25 provinces, *t* = 1, 2, …, 4 waves, where α_*i*_ represents the participant fixed effects, λ_*j*_ represents the province fixed-effects, and *u*_*ijt*_ represents the within-person and within-province residuals.

### Results

The data analysis was conducted using Stata 17.0. The results of the fixed-effects panel model testing the statistical effects of perceived economic inequality and objective economic inequality on sleep quality are presented in Table 1. Sleep quality, Gini coefficients and perceived economic inequality were standardized. Since GDP per capita and household income are right-skewed, we log-transformed these data to achieve a normal distribution and linearize complex economic relationships [57, 64].

The analysis revealed no significant association between objective economic inequality and sleep quality, β = -0.006, *SE* = 0.008, *t* = -0.76, *p* = .456. Contrary to Hypothesis 1a, changes in objective inequality within provinces over time did not predict changes in individual sleep quality. However, higher perceived economic inequality predicted poorer sleep quality, β = -0.011, *SE* = 0.005, *t* = -2.21, *p* = .037. Consistent with Hypothesis 1b, a one-unit increase in perceived inequality over time for a given participant is associated with approximately 0.01-unit decrease in sleep quality per year.

We also tested perceived inequality and the Gini index as individual predictors in separate models, and conducted sensitivity analyses excluding provinces with small numbers of observations, with the findings remaining consistent across all specifications (see *Supplementary Materials*). Moreover, there was no significant difference in the associations between economic inequality (both objective and perceived) and sleep quality across different SES groups (see *Supplementary Materials*).

**Table 1:** Study 1: results from the Fixed-Effects panel model testing the associations between inequality and sleep quality

Sleep quality	β	SE	t-ratio	*p*	95% CI	
Gini	-0.006	0.008	-0.76	.456	[-0.022	0.010]
Perceived economic inequality	-0.011	0.005	-2.21	.037	[-0.021	-0.001]
Age	-0.023	0.007	-3.29	.003	[-0.038	-0.009]
Education	-0.022	0.049	-0.46	.650	[-0.123	0.078]
Household annual income	0.011	0.015	0.75	.460	[-0.020	0.042]
GDP per capita	0.107	0.282	0.38	.709	[-0.477	0.690]
Year of measurement	-0.007	0.012	-0.62	.538	[-0.031	0.017]

Note. The regression model was estimated with 100,536 observations from 33,122 Chinese participants. *SE* = standard error, CI = Confidence Interval. Note that 89.25% of participants exhibit within-subject variation in perceived inequality over time, and caution is advised when interpreting the results

### Discussion

Study 1 showed that perceived economic inequality predicted poorer sleep quality across different SES groups. In contrast, objective economic inequality did not predict sleep quality. These findings raise an important question about the relative influence of subjective versus objective measures of economic inequality on health, an issue to which we return in the General Discussion section.

Although the findings revealed a negative association between perceived economic inequality and sleep quality, Study 1 did not elucidate *how* perceived economic inequality affects sleep. In Study 2, we examine two psychological mechanisms underlying the association between perceived economic inequality and sleep quality: social comparison and stress. In addition, given that the CFPS uses a single-item indicator of sleep quality, Study 2 includes an established scale to assess sleep quality.

## Studies 2a and 2b

Study 2 used two cross-sectional datasets to replicate the relationship between perceived economic inequality and sleep quality (Hypothesis 1b), while investigating whether social comparison and stress function as psychological mechanisms (Hypothesis 2). Study 2a involved a sample of college students, while Study 2b involved a community sample to test whether the findings could generalize to the general population. Although the studies used different samples, their measures and statistical strategies were highly consistent.

### Method

#### Participants

Study 2a drew on data from a project investigating the effect of childhood family environment on psychosocial adjustment in emerging adulthood. The study was conducted in a Chinese college in November 2022, and involved 668 undergraduates with an average age of 19.96 (*SD* = 1.25), 66.8% of whom were female. The median annual household income was 120,000 CNY (approximately 16,529 USD).

Study 2b was conducted in a community in July 2021, in Guangdong Province, and involved 1,009 community respondents with an average age of 39.84 (*SD* = 10.19), 61.6% of whom were female. Among the respondents, 99.90% had received some levels of education, with 86.81% having completed high school. The median annual household income of the respondents was 130,000 CNY (approximately 17,906 USD). Study 2a (SSDPP-HSC 2022004) and Study 2b (SSDPP-HSC 2022008) received ethical approvals from the institutional review board of the last author’s university. Both studies were conducted in full accordance with the Declaration of Helsinki.

#### Measures

The descriptive statistics for the focal continuous variables can be found in Table S7. For each multi-item measure, the items were averaged. Unless otherwise noted, all response scales ranged from 1 = *strongly disagree/not at all* to 5 = *strongly agree/very much*, with higher mean scores reflecting greater agreement. The questionnaire, including all scales and their respective items, is available on the OSF: https://osf.io/n3fg9/?view_only=49ed37edd68849d78a95a9231fe9952c.

**Perceived Economic Inequality** was assessed using the 4-item subjective economic inequality subscale from the Subjective Inequality Scale (e.g., “Almost all the money that is earned goes to only a few people” [65]; α = 0.79 in Study 2a; α = 0.88 in Study 2b).

#### Psychological mechanisms

***Upward Social Comparison*** was assessed using the 3-item measure of upward social comparison (e.g., I am the kind of person who often compares with the rich [66]; α = 0.91 in Study 2a; α = 0.92 in Study 2b).

***Stress*** was assessed using the 7-item stress subscale of the Depression Anxiety and Stress Scale 21 (DASS-21 [67]; e.g., “I find it hard to wind down”). Respondents reported the frequency with which they experienced stress based on their emotions over the past week form 1 = *not fit at all* to 4 = *very fit* (α = 0.87 in Study 2a; α = 0.88 in Study 2b).

**Sleep Quality** was assessed using the 7-item Insomnia Severity Index (ISI [68]) to assess subjective sleep quality [69, 70]. The Chinese version has been demonstrated satisfactory reliability and validity [71].

The ISI comprises three factors: (i) the severity of nighttime sleep difficulties, (ii) sleep satisfaction, and (iii) the daytime impact of insomnia. Nighttime sleep difficulty was measured using three items (e.g., “Difficulty falling asleep”; α = 0.71 in Study 2a; α = 0.80 in Study 2b). Sleep satisfaction was measured using one item (i.e., “How satisfied are you with your current sleep situation?”).^3^ Impact of insomnia was measured using three items (e.g., “To what extent do you consider your sleep problems interfere with your daily functioning?” α = 0.88 in Study 2b).^4^ Reversed scoring was applied in nighttime sleep difficulty and impact of insomnia so that higher scores reflect better sleep quality. These three indicators formed latent variables for sleep quality.

**Sociodemographic Covariates.** We controlled for age, gender (1 = *male*, 2 = *female*) and SES as covariates. Both objective and subjective SES were taken into account. Objective SES was assessed based on household income. Subjective SES was measured using a ten-rung ladder [72], requiring respondents to indicate their perceived position in society relative to others. A higher rung indicates a higher subjective SES.

#### Statistical strategy

We tested our hypotheses using structural equation modeling (SEM) with Mplus 8.3 [73]^5^. In our model, perceived economic inequality was hypothesized to negatively predict the latent variable of sleep quality (H1b), with sequential mediation through upward social comparison and stress (H2). Missing data were handled using the full information maximum likelihood method. Model fit was assessed using the Comparative Fit Index (CFI), the Root Mean Square Error of Approximation (RMSEA), and the Standardized Root Mean Square Residual (SRMR), with CFI ≥ 0.90, RMSEA < 0.08, and SRMR < 0.08 considered acceptable [75]. To assess the statistical significance of indirect effects, we used the bootstrap method with 10,000 resamples [76]. All models controlled for sociodemographic variables. We also examined the results of upward social comparison and stress as separate mediators (see *Supplementary Materials*).

### Results

**Fig. 1:**
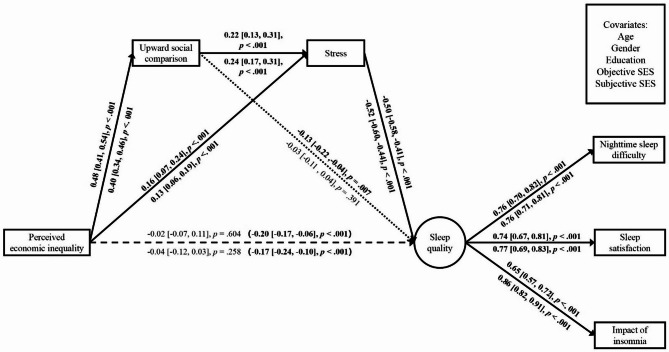
Studies 2a and 2b: Results of Mediation Model Predicting Sleep Quality Controlling for Covariates. *Note.* Study 2a statistics are above line; Study 2b statistics are below line. All coefficients are standardized; numbers in brackets reflect 95% confidence intervals and round brackets reflect the total effect of perceived inequality on sleep quality. Significant paths are in bold; dashed lines reflect non-significant paths; dotted lines reflect mixed paths (i.e., significant in one study only)

The model showed accepted model fit in Studies 2a and 2b (see Table S8). Figure 1 presents the statistical results in Studies 2a and 2b. First, consistent with H1b and replicating Study 1, there was a negative total effect of perceived inequality on the latent factor of sleep quality in Studies 2a and 2b. Second, this association was sequentially mediated by upward social comparison and stress in both studies. Consistent with H2, perceived inequality was positively associated with upward comparison, which in turn was positively associated with stress, with stress negatively predicting sleep quality. Third, the direct effects of perceived inequality on sleep quality were no longer significant once the mediators were taken into account. The indirect effects from perceived inequality to sleep quality via upward comparison and/or stress were significant (except the indirect effect through stress in Study 2b), providing further evidence of the proposed sequential mediation (see Table S9).

### Discussion

Studies 2a and 2b replicated the negative association between perceived economic inequality and sleep quality and examined underlying mechanisms using a college sample and a community sample, respectively. We found that social comparison and stress sequentially mediated the relationship between perceived economic inequality and sleep quality.

## General discussion

Although economic inequality has long been argued to exert adverse effects on mental health [77], few studies have explored how sleep—a factor closely related to mental health—is linked to economic inequality. The current research investigated the relationship between economic inequality and sleep quality, while examining the underlying psychological mechanisms at play.

### Perceived economic inequality is associated with poorer sleep quality

Study 1 revealed that perceived economic inequality is longitudinally associated with poorer sleep quality. That is, a one-unit increase in perceived inequality within individuals is associated with an approximately 0.01-unit decline in their annual sleep quality. This negative link aligns with previous findings that perceived economic inequality tends to worsen mental health [65, 66]. Furthermore, in societies with pronounced economic inequality, both mortality rates increase and lifespans shorten [78] (for more nuanced perspectives, see Chetty et al. [79]). Importantly, it is well-established that insufficient sleep duration and poor sleep quality are linked to higher mortality risk [80]. Given these connections, it is plausible that economic inequality may contribute to poorer mental and physical health by reducing sleep quality.

This study is the first, to our knowledge, to longitudinally investigate the relationship between perceived inequality and sleep quality. This aligns with recent calls to examine the unique associative effects of perceived inequality on various outcomes [12, 27, 81]. It is important to note that the coefficient estimate of perceived economic inequality associated with sleep quality in Study 1 is quite small (β ≈ -0.01), contrasting with the largest estimates in Study 2 (β ≈ -0.20). However, significant but small within-participant effects should not be dismissed as trivial [82], and a modest effect can still have important societal consequences when applying to a large number of people [83, 84]. Arguably, the increasing levels of economic inequality perceived by individuals over a long timeframe may be associated with a practically significant risk to sleep quality at the population level. Moreover, this small effect size in Study 1 may be partially explained by our use of fixed effects models, which eliminate between-individual variation and rely solely on within-individual changes over time. As Mummolo and Peterson note [85], fixed effects estimators substantially reduce the effective variation in the independent variable, often resulting in smaller coefficient estimates. In our case, the within-person variation (Study 1) in perceived inequality is likely considerably smaller than the overall variation (Study 2) in the sample, which would naturally yield more modest effect estimates. This suggests that even small coefficients may represent meaningful within-person associations that occur in the relatively narrow range of variation individuals typically experience in their perceptions of inequality over time. Therefore, these results should be interpreted with caution.

We also acknowledge a limitation in our analysis of the relationship between perceived inequality and sleep quality. When excluding data from the 2020 wave, which coincided with the COVID-19 pandemic, the effect of perceived inequality on sleep quality became non-significant while maintaining the same direction (see Table S6). This sensitivity could be attributed to either reduced statistical power from omitting approximately 23% of observations (which particularly diminishes our ability to detect within-person variations), or potential pandemic-related influences on inequality perceptions and their psychological impacts. Prior research has suggested that the COVID-19 pandemic can heighten the existing inequalities [52]. While our model partials out the effect of time and employs individual and provincial fixed effects, the pandemic’s unique influence on inequality perceptions and sleep quality remains a consideration. Future research specifically designed to examine how major societal events moderate the inequality-sleep relationship would be valuable.

In our research, objective economic inequality was not found to predict sleep quality. This inconsistency between objective and perceived inequality may be partially explained by two factors: First, while the Gini coefficient is the primary measure of objective economic inequality [86], some scholars think the singular focus on it may be insufficient to fully capture the complex nature of inequality and its societal impacts [87]. Second, objective indicators of inequality do not reflect the lived experiences of individuals who face inequality daily, and that subjective indicators may be more closely related to these experiences [27, 81, 88]. In other words, objective indicators might be noisier and more detached from individuals’ actual experiences.

Furthermore, past research indicates that the relationship between inequality and well-being largely depends on how individuals interpret that inequality [89, 90]. When individuals perceive high social mobility or believe in equal opportunities in the context of high inequality, the negative association between inequality and well-being is reduced. That is, perceived inequality reflects individuals’ cognitive responses to inequality more directly, potentially leading to a stronger connection with sleep quality. In contrast, objective inequality is more likely affect well-being in conjunction with other psychological constructs [56]. If people strongly believe in high social mobility in the context of high inequality, the negative association between objective inequality and sleep quality could be lessened. Future research should explore a broader range of objective indicators and adopt a multidimensional approach to studying economic inequality.

It is worth noting that as provincial-level Gini coefficients from administrative data are unavailable in China, researchers are left to rely on existing survey data for inequality estimates [55]. In this study, we used the CFPS—the largest representative national datasets in China—to calculate Gini coefficients for each province, which yielded results similar to those reported by Zhang and Churchill [91]. While we have made every effort to compute the most accurate provincial Gini coefficients possible within our constraints, we acknowledge that estimates derived from secondary data may inevitably differ from the true values. Furthermore, different datasets and calculation methods will produce varying Gini coefficients [92]. Therefore, interpretation of these Gini coefficients should be approached with caution.

### Upward social comparison and stress as psychological mechanisms

Study 2 replicates and extends the findings from Study 1, revealing that upward social comparison and stress can explain the link between perceived inequality and sleep quality. Our research aligns with prior work showing that people perceiving higher economic inequality tend to compare themselves to those who are wealthier, increasing the risk of psychological health problems [34, 66]. The current study advances this line of research by demonstrating that perceived economic inequality is associated with upward social comparison, which is in turn related to poorer sleep quality.

Furthermore, building on prior research linking good sleep quality to positive affect [93] and poor sleep quality to increased aggression, depression, and anxiety [94], our findings support the notion that stress stemming from economic inequality is linked to poorer mental health [43, 95]. This is consistent with the status anxiety hypothesis, which posits that inequality can lead to stress and anxiety about one’s social standing [13].

Nevertheless, the existing findings are not always conclusive as some scholars have expressed skepticism towards the status anxiety hypothesis [42]. By focusing on perceived inequality rather than actual inequality, and its relationship with sleep quality rather than general mental disorders, our research offers a nuanced test of this hypothesis, thereby significantly contributing to advancing the ongoing debate in this area.

More importantly, the current research reveals a sequential mediation that perceived inequality is associated with upward social comparison, which is in turn linked to increased stress, and ultimately related to poorer sleep quality. This nuanced understanding of the underlying mechanisms enhances our knowledge of the psychosocial consequences of inequality, highlighting the interplay between upward comparison and stress in explaining the relationship between inequality and sleep. These findings underscore the need for multi-faceted interventions targeting both upward social comparison and stress management (e.g., shift-and-persist strategies [96]) among those facing higher levels of perceived inequality.

We observed similar path model results in both university students and community residents, indicating the generalizability of our findings. However, it’s worth noting that these groups may perceive economic inequality differently. As Schmalor and Heine noted [65], perceptions of economic inequality can vary by demographic backgrounds (e.g., country). Although we used the same upward comparison measures in both college and community samples, college students typically do not have jobs or salaries and may not perceive economic inequality as strongly as community residents. While the current findings revealed similar patterns between college students and community residents, the two groups may differ in their cognitive estimations of economic inequality—both in terms of its extent and perceived sources. These differences warrant further investigations in future research.

The current study did not examine the role of downward social comparison in the relationship between economic inequality and sleep quality, as longitudinal evidence [66] indicates that downward comparison does not directly predict well-being. While existing research has shown that inequality primarily predicts upward comparison rather than downward comparison, as it offers more opportunities to compare oneself to those who are better off [40], we acknowledge that inequality simultaneously provides opportunities for downward comparison [66]. Although less emphasized in our research framework, downward comparison can serve as an important defensive mechanism against self-evaluative threats, particularly for individuals experiencing various stressors [97, 98]. This suggests that inequality, while often perceived negatively, could paradoxically provide individuals with opportunities to restore their self-esteem. This is a provocative hypothesis worth exploring in future research.

### Limitations and future directions

The current study has several limitations. First, the assessment of sleep quality relied on self-reported measures, which may introduce a bias [99]. Future research could benefit from combining subjective and objective assessment methods, such as actigraphy, to gather more comprehensive sleep data [100]. Specifically, these methods could be used to assess total sleep time (minutes), sleep efficiency (defined as total time asleep out of total time in bed), total number of nighttime awakenings, and total minutes awake during the night [101]. This approach would provide a more accurate appraisal of individuals’ sleep status.

Second, Study 2 used cross-sectional data to investigate the psychological mechanisms underlying inequality and sleep, providing preliminary insights into this complex association. Furthermore, both samples in Study 2 were geographically limited—one comprising students from Guangdong Province and the other consisting of community residents from Zhuhai City only. This may potentially limit generalizability to the broader Chinese population. Previous research highlighted that culture moderates the relationship between economic inequality and well-being [102]. In collectivist cultures like China, economic inequality may have a stronger negative effect on well-being due to the emphasis on social harmony and group welfare. In contrast, individualistic cultures, such as the U.S., may view economic inequality as a reflection of personal achievement, leading to a weaker negative impact on health. Thus, the negative relationship between perceived inequality and sleep quality observed in our study needs further exploration in individualistic contexts. The use of longitudinal data and diverse samples is recommended [103–105] in future research to achieve a more precise understanding of the causal dynamics involved.

Finally, the current study highlights just one potential pathway of the association between economic inequality and sleep quality, and uses a general stress measure that may capture numerous sources of stress, which may not be limited to financial stress related to upward comparison. Therefore, future research should consider employing more refined stress measurement tools to better delineate the mechanisms through which economic inequality impacts sleep quality. Additionally, while our model demonstrates a significant relationship between economic inequality and stress through upward comparison, it is important to note that this relationship may not be limited solely to this process. Economic inequality can also influence other psychological responses, such as upward mobility expectations [106] or economic goals [107]. Additionally, it weakens social ties [77] (but see Kim et al. [108]), potentially leading individuals to withdraw from social interactions [109] (but may prioritize close family ties, see Fernandez-Urbano [110]). This may further increase feelings of stress or even loneliness and affect sleep patterns [111]. Furthermore, (subjective) physical health may also play a role in this relationship, potentially moderating the relationship between economic inequality and sleep quality [112]. Future research should explore other psychological and social mechanisms to fully understand the link between inequality and sleep.

## Conclusion

This work aimed to reveal the relationship between economic inequality and sleep quality and to uncover the underlying mechanisms in this relationship. Our study demonstrated a long-term within-participant detrimental link between perceived economic inequality and sleep quality. Furthermore, we found that upward social comparison and stress accounted for the effect of economic inequality on sleep quality. These findings underscore the importance of promoting shared prosperity to mitigate perceptions of inequality and enhance well-being of population.

## Supplementary Information

Below is the link to the electronic supplementary material.

Supplementary Material 1

## Data Availability

Complete materials, raw data (or instructions to retrieve the secondary survey data), and scripts reproducing the findings are available via the OSF: https://osf.io/n3fg9/.

## Footnotes

1 The CFPS tracks family members within households, with variations in the number of respondents across waves due to deaths, lost contact, family separation, and family remarriage (resulting in new respondents). Therefore, T1 involved 35,719 respondents; T2 had 36,892 respondents; T3 had 37,354 respondents, and T4 had 28,590 respondents. To take advantage of the longitudinal nature of the data, we removed respondents who only participated in one wave (singleton data). Given the exclusion of singleton respondents, the risk of bias from non-random attrition is a concern. To address this, we compared sleep quality and perceived inequality between singletons and those who participated in multiple waves. An independent samples *t*-test revealed significant differences between singleton (*M* = 3.27, *SD* = 0.90) and non-singleton (*M* = 3.32, *SD* = 0.86) participants in sleep quality, *t* = -5.26, *p* < .001, but not in perceived inequality, *t* = 1.50, *p* = .134. A regression analysis with attrition status as the predictor explained only 0.1% of the variance in sleep quality. Although attrition was not completely random with respect to sleep quality, the minimal variance explained (R² = 0.001) indicates that potential bias is unlikely to meaningfully affect our findings.

2 Fixed-effects panel model is a commonly used method for panel data analysis, which is similar to traditional regression but incorporates *N* – 1 participant-specific dummy variables. As such, this method eliminates all observable and unobservable wave-invariant differences between participants, allowing one to focus exclusively on changes within participants over time [61]. Imai and Kim [62] referred to fixed-effects panel model as the “default method for estimating causal effects using panel data” and Hill et al. [63] noted that “the fixed-effects model for panel data is now widely recognized as a powerful analytical tool for longitudinal data analysis.”

3 In Study 2b, respondents answered this item from 0 = *very much* to 4 = *not at all*, and the scoring was reversed in subsequent analysis.

4 In Study 2a, due to a technical error, the questionnaire omitted an item measuring the impact of insomnia (“How worried/distressed are you about your current sleep problem?”). Consequently, the analysis only used data of the remaining two items.

5 In terms of statistical power, the ratio of participants to parameters (*n* = 11) was approximately 61:1 in Study 2a, and 92:1 in Study 2b, exceeding the recommended 20:1 ratio [74]. The ample sample sizes ensured adequate power to test our hypothesized path model.

## Declarations

**Clinical trial**: Clinical trial number: Not applicable, as this study was not conducted in a clinical setting.

## Abbreviations

CFI: Comparative fit index
CI: Confidence Interval
GDP: Gross Domestic Product
ISI: Insomnia Severity Index
RMSEA: Root Mean Square Error of Approximation
SD: Standard Deviation
SEM: Structural Equation Modeling
SES: Socioeconomic Status
SRMR: Standardized Root Mean Square Residual
