# Supplementary material for: Perceived economic inequality is linked to poorer sleep quality
Source: BMC Psychol. 2025 Oct 1;13:1099. doi: 10.1186/s40359-025-03405-5 (PMC12487066; doi:10.1186/s40359-025-03405-5)
Supplement: Supplementary file 1 — Supplementary Material 1 [file 40359_2025_3405_MOESM1_ESM.docx]

Supplementary Materials for

Perceived Economic Inequality is Linked to Poorer Sleep Quality

**This file includes:**

**Study 1**

Table S12

The Description of the Household Sample Size in Each Province3

Table S24

Additional Analysis in Study 15

Table S37

Table S38

**Study 2**

Table S49

Table S510

Table S611

Additional Analysis in Study 212

Table S714

Table S814

Figure S115

Figure S216

**Reference**17

| **Table S1**  *Study 1: Descriptive Statistics for the Focal Variables* | | | | |
| --- | --- | --- | --- | --- |
| Variables | Mean | *SD* | Min | Max |
| Gini | 0.49 | 0.05 | 0.37 | 0.67 |
| Perceived economic inequality | 6.83 | 2.48 | 0 | 10 |
| Sleep quality | 3.24 | 0.91 | 1 | 4 |
| Age | 46.15 | 16.24 | 16 | 99 |
| Education | 2.75 | 1.40 | 1 | 8 |
| Household annual income* | 89,632.88 | 172,691.80 | 1 | 12,445,723 |
| GDP per capita | 53,377.35 | 27,482.97 | 18,947 | 164,158 |
| *Note.* *The household annual income has been adjusted for annual inflation to 2020 CNY and was equivalized using OECD’s square root approach. The Gini coefficients represent province-level income inequality calculated from CFPS household income data. The wide range (0.37-0.67) reflects the substantial regional economic disparities across Chinese provinces. While these values should be interpreted with appropriate caution due to sampling limitations, they capture meaningful variation in province-level inequality consistent with China’s uneven economic development patterns. | | | | |

**The Description of the Household Sample Size in Each Province**

We calculated the median, minimum and maximum number of households in each province for the years 2012, 2016, 2018, and 2020. The description of the household sample size in each province as shown in **Table S2**.

While these samples cannot perfectly represent the true provincial population, they represent the most representative income data *available* at the provincial level. We acknowledge that provinces with smaller samples (e.g., Tianjin, Beijing) may have less precise inequality estimates. To address this limitation, we conducted sensitivity analyses excluding provinces with fewer than 200 households (i.e., Beijing, Fujian, Tianjin, Chongqing), and the main findings remained consistent. Specifically, the analysis revealed no significant association between objective economic inequality and sleep quality, β = -0.008, *SE* = 0.008, *t* = -0.93, *p* =.364. Contrary to Hypothesis 1a, residents in more unequal provinces did not report poorer sleep quality than those in less unequal provinces. However, higher perceived economic inequality predicted poorer sleep quality, β = -0.010, *SE* = 0.005, *t* = -2.19, *p* =.041. Consistent with Hypothesis 1b, a one-unit increase on perceived inequality over time for a given participant is associated with approximately 0.01 decrease on sleep quality per year.

**Table S2**

*Study 1: The Description of the Household Sample Size in Each Province*

| **Provinces** | **Median** | **Min** | **Max** |
| --- | --- | --- | --- |
| Anhui | 283 | 232 | 295 |
| Beijing | 129 | 77 | 155 |
| Chongqing | 157 | 109 | 164 |
| Fujian | 176 | 150 | 188 |
| Gansu | 1,511 | 1,333 | 1,595 |
| Guangdong | 1,260 | 1,187 | 1,465 |
| Guangxi | 275 | 230 | 299 |
| Guizhou | 421 | 321 | 431 |
| Hebei | 747 | 727 | 790 |
| Henan | 1,487 | 1,209 | 1,518 |
| Heilongjiang | 419 | 399 | 446 |
| Hubei | 222 | 171 | 232 |
| Hunan | 411 | 313 | 434 |
| Jilin | 274 | 234 | 277 |
| Jiangsu | 286 | 264 | 313 |
| Jiangxi | 273 | 221 | 284 |
| Liaoning | 1,326 | 1,026 | 1,378 |
| Shandong | 692 | 651 | 712 |
| Shanxi | 574 | 546 | 605 |
| Shaanxi | 301 | 282 | 327 |
| Shanghai | 887 | 648 | 1,039 |
| Sichuan | 659 | 532 | 724 |
| Tianjin | 92 | 81 | 105 |
| Yunnan | 370 | 341 | 408 |
| Zhejiang | 279 | 227 | 347 |
| *Note.* ‘Median’ refers to the median number of households per province, ‘Min’ refers to the minimum number of households per province, and ‘Max’ refers to the maximum number of households per province. | | | |

**Table S3**

*Study 1: Gini Coefficient for Each Province Across Four Waves*

| **Provinces** | **2012** | **2016** | **2018** | **2020** |
| --- | --- | --- | --- | --- |
| Anhui | 0.48 | 0.56 | 0.50 | 0.49 |
| Beijing | 0.40 | 0.46 | 0.51 | 0.54 |
| Chongqing | 0.49 | 0.45 | 0.46 | 0.53 |
| Fujian | 0.52 | 0.65 | 0.50 | 0.49 |
| Gansu | 0.50 | 0.43 | 0.46 | 0.43 |
| Guangdong | 0.51 | 0.53 | 0.49 | 0.54 |
| Guangxi | 0.52 | 0.55 | 0.46 | 0.42 |
| Guizhou | 0.51 | 0.62 | 0.52 | 0.54 |
| Hebei | 0.50 | 0.45 | 0.49 | 0.47 |
| Henan | 0.50 | 0.45 | 0.46 | 0.42 |
| Heilongjiang | 0.48 | 0.40 | 0.40 | 0.38 |
| Hubei | 0.41 | 0.50 | 0.44 | 0.56 |
| Hunan | 0.54 | 0.48 | 0.47 | 0.44 |
| Jilin | 0.43 | 0.40 | 0.46 | 0.47 |
| Jiangsu | 0.50 | 0.50 | 0.52 | 0.44 |
| Jiangxi | 0.47 | 0.40 | 0.46 | 0.47 |
| Liaoning | 0.48 | 0.49 | 0.47 | 0.44 |
| Shandong | 0.55 | 0.50 | 0.50 | 0.49 |
| Shanxi | 0.56 | 0.67 | 0.46 | 0.46 |
| Shaanxi | 0.49 | 0.46 | 0.56 | 0.63 |
| Shanghai | 0.51 | 0.56 | 0.55 | 0.49 |
| Sichuan | 0.60 | 0.48 | 0.48 | 0.54 |
| Tianjin | 0.39 | 0.53 | 0.38 | 0.37 |
| Yunnan | 0.53 | 0.47 | 0.45 | 0.48 |
| Zhejiang | 0.54 | 0.43 | 0.49 | 0.43 |
| *Note.* Gini coefficients were calculated using household income data from the China Family Panel Studies. | | | | |

**Additional Analysis in Study 1**

## Analytical Strategy

We performed two additional fixed-effects panel regression analyses, both of which incorporated participant and province fixed effects. In the first analysis, we regressed annual individual sleep quality on annual individual perceptions of economic inequality:

|  | Sleep Quality_ijt_ = β_0_ + β_1_ Perceived Inequality_ijt_ + β_2_ GDP per capita _ijt_ + β_3_ Age_ijt_ *+* β_4_ Education_ijt_ *+* β_5_ Income_ijt_ *+* Β_6_ Year_ijt_ *+* α*_i_ +* λ*_j_ + u_ijt_* | Eq. 1 |
| --- | --- | --- |

*…i* = 1, 2, ..., 33,122 participants, *j* = 1, 2, …, 25 provinces, *t* = 1, 2, ..., 4 waves, where α*_i_* represents the participant fixed effects, λ*_j_* represents the province fixed-effects, and *u_ijt_* represents the within-person and within-province residuals.

In the second analysis, we regressed annual individual sleep quality on annual province economic inequality:

|  | Sleep Quality_ijt_ = β_0_ + β_1_ Gini_ijt_ + β_2_ GDP per capita _ijt_ + β_3_ Age_ijt_ *+* β_4_ Education_ijt_ *+* β_5_ Income_ijt_ *+* Β_6_ Year_ijt_ *+* α*_i_ +* λ*_j_ + u_ijt_* | Eq. 2 |
| --- | --- | --- |

*…i* = 1, 2, ..., 33,122 participants, *j* = 1, 2, …, 25 provinces, *t* = 1, 2, ..., 4 waves, where α*_i_* represents the participant fixed effects, λ*_j_* represents the province fixed-effects, and *u_ijt_* represents the within-person and within-province residuals.

## Results

The first fixed-effects panel model revealed that perceived inequality was a negative predictor, β = -0.011, *SE* = 0.005, *t* = -2.2, *p* = .037 (see **Table S4**). The second fixed-effects panel model, which did not include perceived inequality, revealed that the Gini index was not also a significant predictor, β = -0.006, *SE* = 0.008, *t* = -0.75, *p* = .463 (see **Table S5**). These results are similar to those obtained when both are considered together.

## Additional Results

Previous research suggests that the association between economic inequality and psychological health is stronger among individuals with low SES than individuals with high SES (Oishi et al., 2011; Sommet et al., 2018). We therefore conducted exploratory analyses to investigate whether the association between inequality and sleep quality differs across SES groups. Specifically, we used inflation-adjusted equivalized household income as an objective SES indicator, and examined the interaction between each measure of economic inequality and SES using the double-demeaning procedure (Giesselmann & Schmidt-Catran, 2022). The interaction between objective economic inequality and SES was not significant, β = 0.020, *SE* = 0.017, *t* = 1.17, *p* = .253. Moreover, the interaction between perceived economic inequality and SES was not significant, β = -0.007, *SE* = 0.017, *t* = 0.44, *p* = .662, suggesting that the negative association between perceived economic inequality and sleep quality did not differ across SES groups.

**Table S4**

*Study 1: Results from the Fixed-Effects Panel Model Testing the Associations Between Perceived Economic Inequality and Sleep Quality*

| Sleep quality | β | *SE* | *t*-ratio | *p* | 95% CI | |
| --- | --- | --- | --- | --- | --- | --- |
| Perceived economic inequality | -0.011 | 0.005 | -2.20 | .037 | [-0.021 | -0.001] |
| Age | -0.024 | 0.007 | -3.27 | .003 | [-0.038 | -0.009] |
| Education | -0.023 | 0.048 | -0.47 | .643 | [-0.123 | 0.077] |
| Household annual income | 0.011 | 0.015 | 0.74 | .465 | [-0.020 | 0.042] |
| GDP per capita | 0.116 | 0.304 | 0.38 | .707 | [-0.513 | 0.744] |
| Year of measurement | -0.007 | 0.012 | -0.58 | .569 | [-0.031 | 0.018] |
| *Note.* The regression model was estimated with 100,536 observations from 33,122 Chinese participants. *SE* = standard error, CI = Confidence Interval. Note that 89.25% of participants exhibit within-subject variation in perceived inequality over time, and caution is advised when interpreting the results. | | | | | | |

**Table S5**

*Study 1: Results from the Fixed-Effects Panel Model Testing the Associations Between Objective Economic Inequality and Sleep Quality*

| Sleep quality | β | *SE* | *t*-ratio | *p* | 95% CI | |
| --- | --- | --- | --- | --- | --- | --- |
| Gini | -0.006 | 0.008 | -0.75 | .463 | [-0.022 | 0.010] |
| Age | -0.023 | 0.007 | -3.25 | .003 | [-0.038 | -0.008] |
| Education | -0.022 | 0.049 | -0.46 | .652 | [-0.123 | 0.078] |
| Household annual income | 0.011 | 0.015 | 0.75 | .460 | [-0.020 | 0.043] |
| GDP per capita | 0.010 | 0.280 | 0.35 | .726 | [-0.479 | 0.677] |
| Year of measurement | -0.007 | 0.012 | -0.62 | .539 | [-0.031 | 0.017] |
| *Note.* The regression model was estimated with 100,536 observations from 33,122 Chinese participants. *SE* = standard error, CI = Confidence Interval. | | | | | | |

**Table S6**

*Study 1: Fixed-Effects Results Excluding 2020 Wave (COVID-19 Period)*

| Sleep quality | β | *SE* | *t*-ratio | *p* | 95% CI | |
| --- | --- | --- | --- | --- | --- | --- |
| Gini | -0.010 | 0.009 | -1.14 | .265 | [-0.029 | 0.008] |
| Perceived economic inequality | -0.009 | 0.006 | -1.63 | .115 | [-0.021 | 0.002] |
| Age | -0.028 | 0.008 | -3.34 | .003 | [-0.045 | -0.011] |
| Education | -0.020 | 0.052 | -0.38 | .709 | [-0.127 | 0.088] |
| Household annual income | 0.014 | 0.014 | 1.00 | .328 | [-0.014 | 0.042] |
| GDP per capita | 0.300 | 0.356 | 0.84 | .408 | [-0.435 | 1.034] |
| Year of measurement | -0.012 | 0.017 | -0.69 | .494 | [-0.048 | 0.024] |
| *Note.* The regression model was estimated with 77,537 observations from 29,983 Chinese participants. *SE* = standard error, CI = Confidence Interval. Note that 87.76% of participants exhibit within-subject variation in perceived inequality over time, and caution is advised when interpreting the results. | | | | | | |

| **Table S7**  *Studies 2a and 2b: Descriptive Statistics for Each Continuous Variable* | | | | | | | | |
| --- | --- | --- | --- | --- | --- | --- | --- | --- |
|  | Study 2a | | | | Study 2b | | | |
| Variables | Mean | *SD* | Min | Max | Mean | *SD* | Min | Max |
| Perceived economic inequality | 2.80 | 0.83 | 1.00 | 5.00 | 2.52 | 0.87 | 1.00 | 5.00 |
| Upward social comparison | 2.40 | 1.01 | 1.00 | 5.00 | 1.84 | 0.75 | 1.00 | 5.00 |
| Stress | 1.93 | 0.69 | 1.00 | 4.00 | 0.42 | 0.51 | 0 | 2.86 |
| Sleep quality |  |  |  |  |  |  |  |  |
| Nighttime sleep difficulty | 4.26 | 0.74 | 1.00 | 5.00 | 3.52 | 0.64 | 0 | 4.00 |
| Sleep satisfaction | 3.36 | 1.00 | 1.00 | 5.00 | 2.84 | 1.10 | 0 | 4.00 |
| Impact of insomnia | 3.25 | 1.04 | 1.00 | 5.00 | 3.30 | 0.86 | 0 | 4.00 |
| Age | 19.96 | 1.25 | 18.00 | 29.00 | 39.84 | 10.19 | 18.00 | 59.00 |
| Education |  |  |  |  | 4.49 | 0.79 | 1.00 | 6.00 |
| Subjective socioeconomic status | 4.95 | 1.36 | 1.00 | 9.00 | 5.31 | 1.69 | 1.00 | 10.00 |
| Objective socioeconomic status* | 4.05 | 0.33 | 3.00 | 5.54 | 5.10 | 0.44 | 0 | 7.30 |
| *Note.* *The objective socioeconomic status was derived from the logarithmically transformed household income. | | | | | | | | |

| **Table S8**  *Studies 2a and 2b: the Fitting Index of Structural Equation Modeling* | | |
| --- | --- | --- |
|  | Study 2a | Study 2b |
| χ² | 103.782 | 89.15 |
| *df* | 26 | 26 |
| χ²/*df* | 3.99 | 3.43 |
| *p* | <.001 | <.001 |
| CFI | 0.922 | 0.960 |
| RMSEA | 0.067 | 0.049 |
| SRMR | 0.037 | 0.031 |

| **Table S9**  *Studies 2a and 2b: Indirect Effects of the Relationship Between Perceived Economic Inequality and Sleep Quality* | | | | | | | |
| --- | --- | --- | --- | --- | --- | --- | --- |
| Indirect Pathway | Study 2a | | | | Study 2b | | |
|  | | β | *p* | 95% CI | β | *p* | 95% CI |
| Perceived inequality → upward comparison → stress | | 0.11 | <.001 | [0.06, 0.15] | 0.10 | .017 | [0.07, 0.13] |
| Perceived inequality → upward comparison → sleep quality | | -0.06 | .007 | [-0.11, -0.02] | -0.01 | .392 | [-0.05, 0.02] |
| Perceived inequality → stress → sleep quality | | -0.08 | .001 | [-0.13, -0.04] | -0.07 | .019 | [-0.11, -0.03] |
| Perceived inequality → upward comparison → stress → sleep quality | | -0.05 | <.001 | [-0.08, -0.03] | -0.05 | .009 | [-0.07, -0.03] |
| *Note*. CI = confidence interval. | | | | | | | |

**Additional Analysis in Study 2**

**Results**

***Upward Social Comparison as Sperate Mediator***

The model showed accepted model fit in Studies 2a and 2b (see **Table S10**). As shown in **Figure S1**, there was a negative total effect of perceived inequality on the latent factor of sleep quality in Studies 2a and 2b. Second, this association was mediated by upward social comparison in both studies. Moreover, perceived inequality was positively associated with upward social comparison, which itself was negatively predicting sleep quality. Third, the direct effects of perceived inequality on sleep quality were no longer significant once the mediators were taken into account. Moreover, the indirect effects of perceived inequality on sleep quality via upward social comparison were significant in Studies 2a (β = -0.11 [-0.16, -0.06], *p* < .001) and 2b (β = -0.06 [-0.06, -0.02], *p* = .001).

***Stress as Sperate Mediator.***

The model showed unaccepted model fit in Study 2a. Despite showing unacceptable model fit indices in Study 2a, we report the specific path results for comparative purposes. In contrast, the model showed acceptable fit in Study 2b (see **Table S11**). As shown in **Figure S2**, there was a negative total effect of perceived inequality on the latent factor of sleep quality in Studies 2a and 2b. Second, this association was mediated by stress in both studies. Moreover, perceived inequality was positively associated with stress, which itself was negatively predicting sleep quality. Third, the direct effects of perceived inequality on sleep quality were no longer significant once the mediators were taken into account. Moreover, the indirect effects of perceived inequality on sleep quality via stress were significant in Studies 2a (β = -0.14 [-0.18, -0.09], *p* < .001) and 2b (β = -0.12 [-0.16, -0.08], *p* < .001).

| **Table S10**  *Studies 2a and 2b: the Fitting Index of Structural Equation Modeling for Upward Social Comparison as Sperate Mediator* | | |
| --- | --- | --- |
|  | Study 2a | Study 2b |
| χ² | 63.162 | 63.456 |
| *df* | 19 | 19 |
| χ²/*df* | 3.32 | 3.34 |
| *p* | <.001 | <.001 |
| CFI | 0.943 | 0.964 |
| RMSEA | 0.059 | 0.048 |
| SRMR | 0.029 | 0.025 |

| **Table S11**  *Studies 2a and 2b: the Fitting Index of Structural Equation Modeling for Stress as Sperate Mediator* | | |
| --- | --- | --- |
|  | Study 2a | Study 2b |
| χ² | 102.314 | 68.276 |
| *df* | 19 | 19 |
| χ²/*df* | 5.38 | 3.59 |
| *p* | <.001 | <.001 |
| CFI | 0.896 | 0.963 |
| RMSEA | 0.081 | 0.051 |
| SRMR | 0.039 | 0.029 |

**Figure S1**

*Studies 2a and 2b: Results of Mediation Model with Upward Social Comparison as Separate Mediator Predicting Sleep Quality Controlling for Covariates*

**
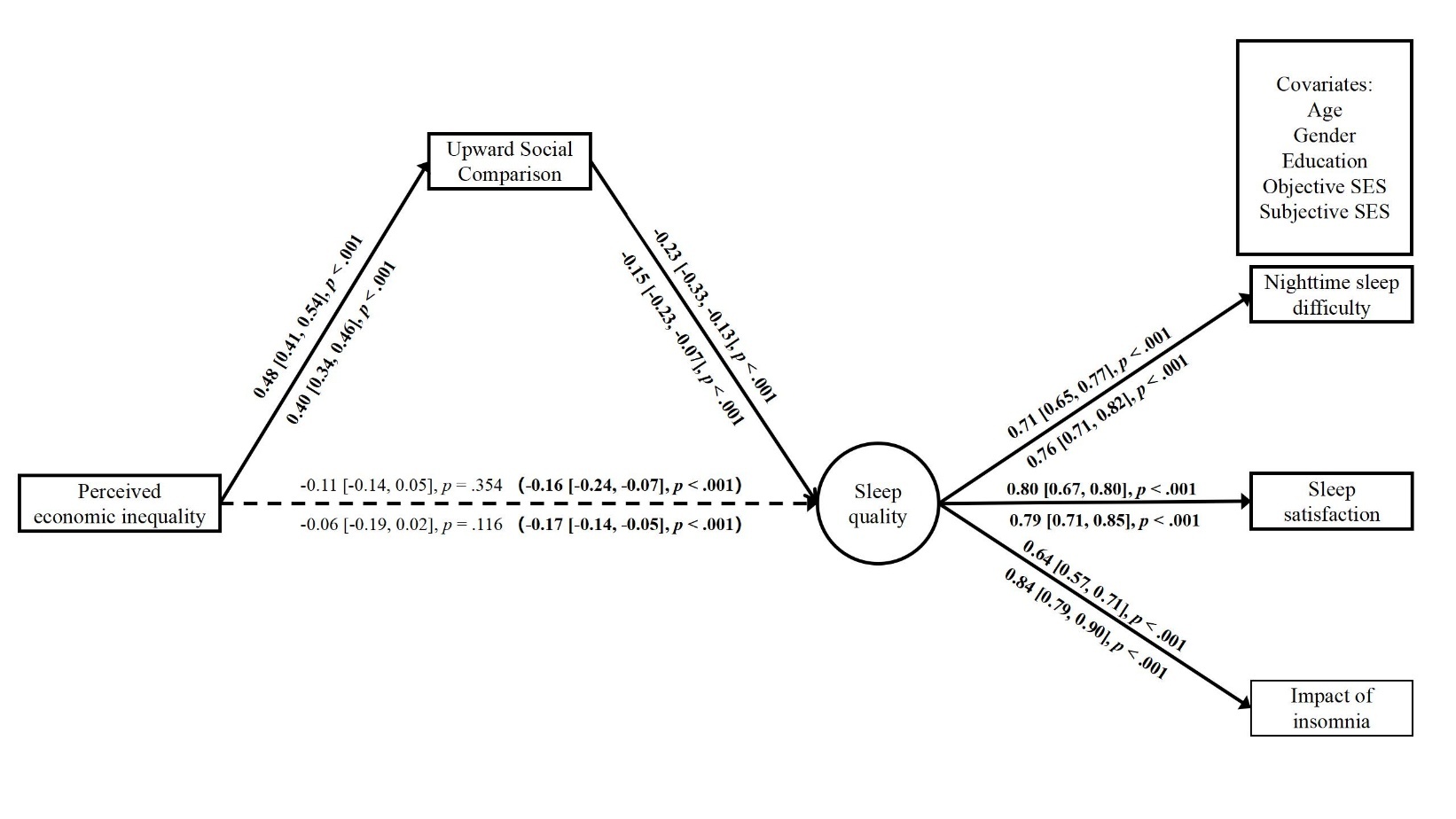
**

*Note.* Study 2a statistics are above line; Study 2b statistics are below line. All coefficients are standardized; numbers in brackets reflect 95% confidence intervals and round brackets reflect the total effect of perceived inequality on sleep quality. Significant paths are in bold; dashed lines reflect non-significant paths.

**Figure S2**

*Studies 2a and 2b: Results of Mediation Model with Stress as Separate Mediator Predicting Sleep Quality Controlling for Covariates*

*
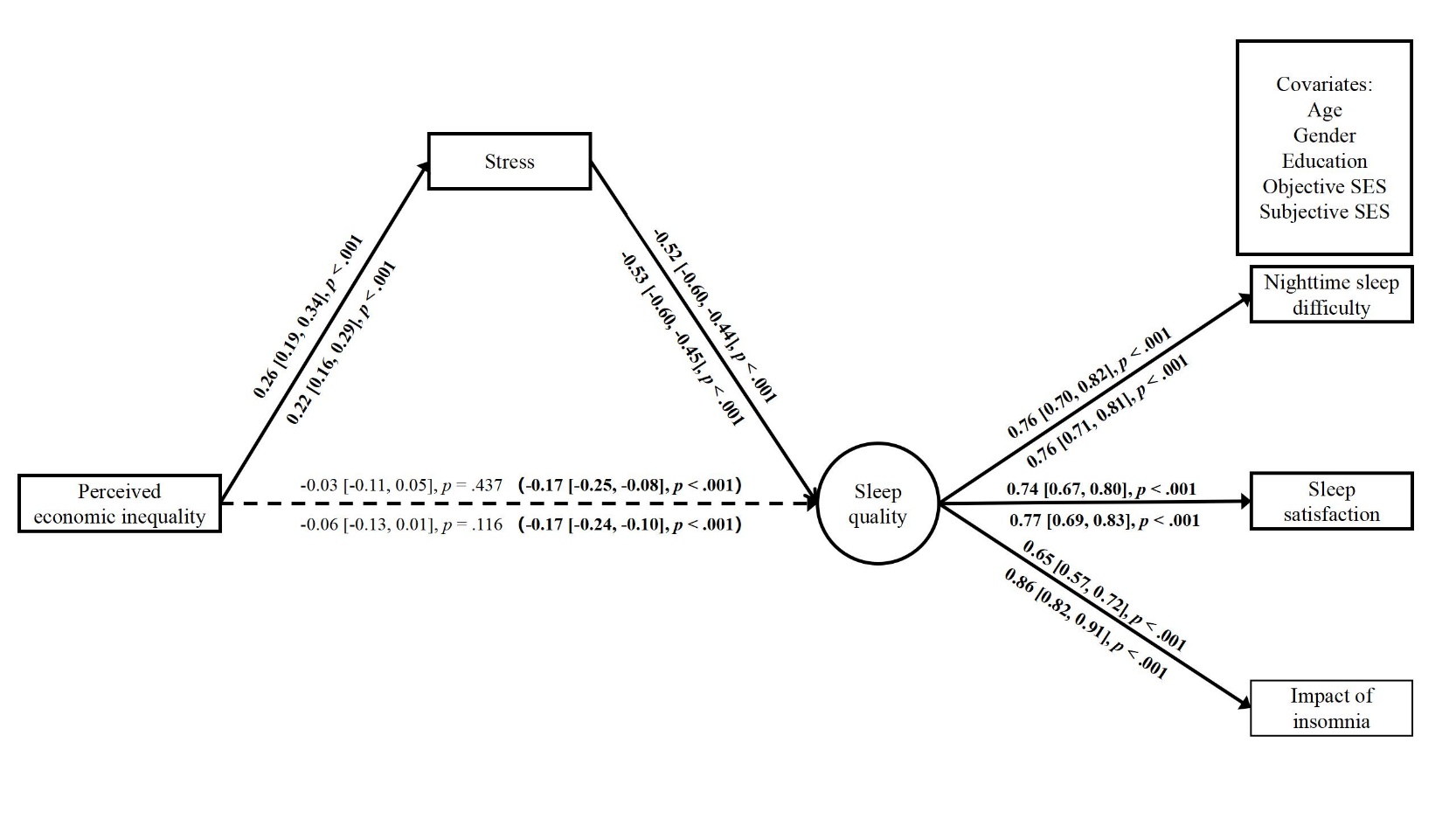
*

*Note.* Study 2a statistics are above line; Study 2b statistics are below line. All coefficients are standardized; numbers in brackets reflect 95% confidence intervals and round brackets reflect the total effect of perceived inequality on sleep quality. Significant paths are in bold; dashed lines reflect non-significant paths.

**References**

Giesselmann, M., & Schmidt-Catran, A. W. (2022). Interactions in fixed effects regression models. *Sociological Methods & Research*, *51*(3), 1100-1127. <https://doi.org/10.1177/0049124120914934>

Oishi, S., Kesebir, S., & Diener, E. (2011). Income inequality and happiness. *Psychological Science*, *22*, 1095-1100. <https://doi.org/10.1177/0956797611417262>

Sommet, N., Morselli, D., & Spini, D. (2018). Income inequality affects the psychological health of only the people facing scarcity. *Psychological Science*, *29*, 1911-1921. <https://doi.org/10.1177/0956797618798620>
